# Plaques en prairie fauchée

**DOI:** 10.1016/j.idcr.2021.e01080

**Published:** 2021-03-23

**Authors:** Eric Farfour, David Zucman

**Affiliations:** a Service de biologie Clinique, Hôpital Foch, 40 rue Worth, 92150, Suresnes, France; b Service de médecine interne, Hôpital Foch, Suresnes, France

**Keywords:** Syphilis, HIV, AIDS, Mowed meadow, Plaque en prairie fauchée, Rash

## Abstract

We report a clinical picture of a mowed meadow pattern of the tongue in a 29-year-old sexually active man who has sex with men. The painful lesions also called "Plaques en prairie fauchée", occur in the context of the discovery of a postive HIV serology. The diagnosis of secondary syphilis was confirmed by a positive syphilis serology (nontreponemal RPR test title of 64) and a positive *Treponema pallidum* PCR, amplifying the tpp47 gene, on a tongue swab. The lesions completely resolve with a penicillin treatment.

## Highlights

• Mowed meadow pattern of the tongue also called “*Plaques en prairie fauchée*” is a manifestation of secondary syphilis.
• It results from the hematogenous dissemination of *Treponema pallidum* from syphilitic chancres.
• Suspected case is confirmed by serology or nucleic acid amplification on a tongue swab.

**Fig. 1:**
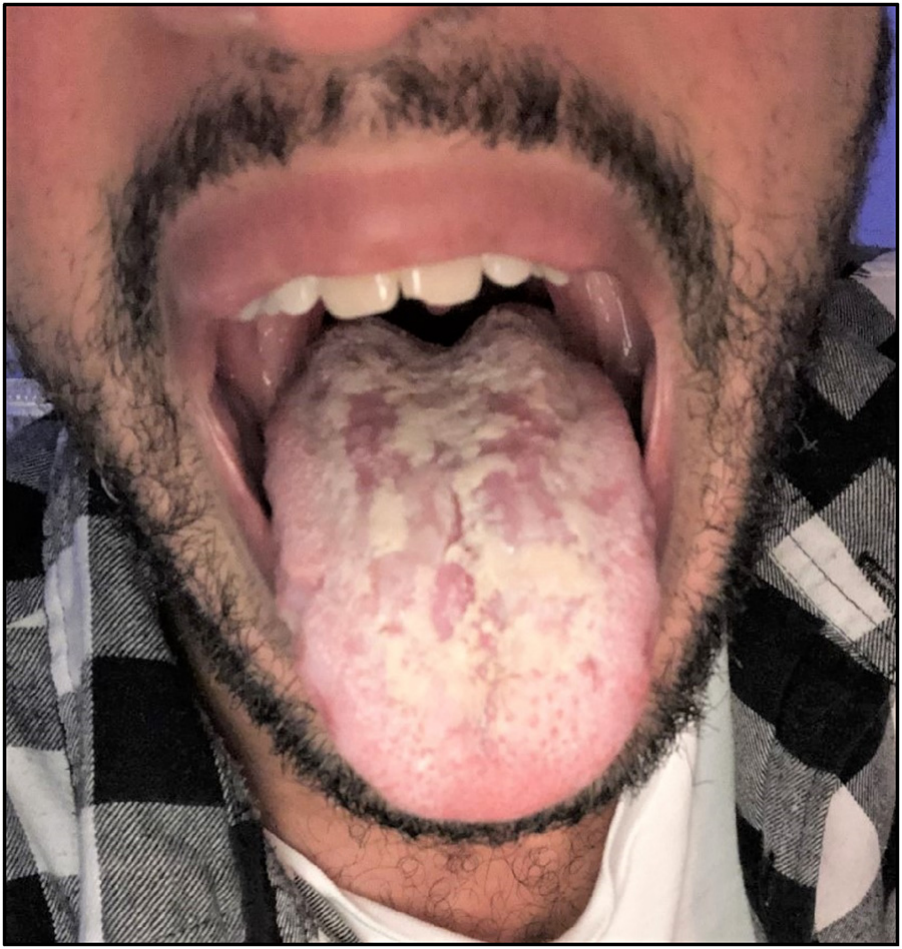
Scattered depapillation of the tongue in a mowed meadow pattern also called “*Plaques en prairie fauchée*”.

A 29-year-old man was referred to a specialized HIV-consultation for the discovery of a positive HIV serology and an unexplained painful lesion of the tongue with a painless maculopapular rash (Fig. 1). The patient is a sexually active man having sex with men (MSM) who used to take HIV-pre-exposition prophylaxis (PreP) until the COVID-19 lockdown in March 2020. He was diagnosed with a mild symptomatic COVID-19 a month earlier, recovering spontaneously before skin and tongue lesions appear gradually. They were first attributed to an immunological reaction to SARS-CoV-2. The patient looked for voluntary HIV testing in the context of multiple unprotected sexual intercourses. On the first HIV-specialized consultation, the examination of the oral cavity shows scattered depapillation of the dorsal side of the tongue. He also presented a maculopapular rash of the entire body including soles and palms. Biological screening of sexually transmitted infections, including syphilis serology was performed [1]. The total syphilis antibodies (Abbott, Alinity I) and the non-treponemal RPR test (title of 64, BioRad) were positive. A *Treponema pallidum* PCR, amplifying the *tpp47* gene, on a tongue swab, was positive. *Neisseria gonorrhea* and *Chlamydia trachomatis* PCR performed on urine, pharyngeal and rectal swabs were all negative. He completely recovered after administration of a single dose of 2.4 million units of intramuscular penicillin.

The incidence of syphilis has strongly increased over the past decade mainly in men who have sex with men (MSM) [2]. The secondary stage of syphilis results from the hematogenous dissemination of *Treponema pallidum* from syphilitic chancres [3]. It usually presents as skin rash and/or mucous lesions occurring 1–3 months after the primary lesion. The most frequent lesions are symmetric painless, nonpruritic papular eruptions involving the entire trunk and the extremities, including the palms of the hands and the soles of the feet. Oral syphilis is the most frequent extragenital location of secondary syphilis [4]. The mowed meadow pattern of the tongue also called “*Plaques en prairie fauchée*” is a typical painful scattered depapillation. It could be associated with a painful split papule of the oral commissure [5]. Differential diagnosis includes other depapillation lesions of the tongue such as geographical tongue. However, the context is evocative of syphilis. The diagnosis is confirmed by serological test and/or a specific PCR of a tongue swab. The lesions usually resolve with penicillin treatment.

## Declaration of Competing Interest

No conflicts of interest.

## Funding

No funding.

## Ethical approval

Written informed consent was obtained from the patient for publication of this case report and accompanying images. A copy of the written consent is available for review by the Editor-in-Chief of this journal on request

## Consent

Informed consent was obtained for publication of this case report and accompanying images.

## CRediT authorship contribution statement

**Eric Farfour:** Writing - original draft. **David Zucman:** Writing - review & editing.
